# A phase‐II randomized controlled pilot study of nicotinamide riboside supplementation in older adults with amnestic mild cognitive impairment

**DOI:** 10.1002/alz.71605

**Published:** 2026-07-21

**Authors:** Christopher R. Martens, Kevin P. Decker, Theodore M. DeConne, Faria Sanjana, Fiona Horvat, Nicholas A. Rizzi, Catherine Awad, Elizabeth Habash, Joshua C. Hobson, Mary K. Kramer, Michael L. Armstrong, Nichole Reisdorph, Ryan T. Pohlig, Alyssa M. Lanzi, Curtis L. Johnson, Matthew L. Cohen, James M. Ellison

**Affiliations:** ^1^ Department of Kinesiology & Applied Physiology University of Delaware Newark Delaware USA; ^2^ Delaware Center for Cognitive Aging Research University of Delaware Newark Delaware USA; ^3^ Department of Internal Medicine Section on Gerontology and Geriatric Medicine Wake Forest University School of Medicine Winston‐Salem North Carolina USA; ^4^ Department of Biomedical Engineering University of Delaware Newark Delaware USA; ^5^ Department of Pharmaceutical Sciences Skaggs School of Pharmacy and Pharmaceutical Sciences University of Colorado Anschutz Medical Campus Denver Colorado USA; ^6^ Department of Epidemiology University of Delaware Newark Delaware USA; ^7^ Department of Communication Sciences & Disorders University of Delaware Newark Delaware USA; ^8^ Department of Psychiatry & Human Behavior Thomas Jefferson University Philadelphia Pennsylvania USA

**Keywords:** cerebral blood flow, clinical trial, mild cognitive impairment, NAD+, nicotinamide riboside

## Abstract

**INTRODUCTION:**

Declining nicotinamide adenine dinucleotide (NAD^+^) may elevate risk of Alzheimer's disease.

**METHODS:**

We conducted a 12‐week double‐blind, randomized, placebo‐controlled pilot study to evaluate the safety, tolerability, and preliminary efficacy of the NAD^+^ precursor, nicotinamide riboside (NR), for enhancing cognitive function and cerebral blood flow (CBF) in adults with amnestic mild cognitive impairment (aMCI).

**RESULTS:**

42 participants completed the study (NR = 22, placebo = 20). Adherence was similar between groups with no serious adverse effects. Blood NAD^+^ increased twofold in the NR group. There were no improvements in cognitive function (primary outcome), total CBF, or blood pressure (secondary outcomes). Exploratory analyses revealed potential increases in regional CBF, particularly in the hippocampus.

**DISCUSSION:**

NR effectively raises NAD^+^ in people with MCI but does not improve cognitive function, total CBF, or blood pressure over 12 weeks. Future studies should investigate regional effects on CBF over longer treatment durations.

## Highlights

- A 12‑week randomized, placebo‑controlled trial of nicotinamide riboside (NR) was conducted in older adults with amnestic mild cognitive impairment (MCI).
- NR supplementation doubled blood nicotinamide adenine dinucleotide (NAD^+^) levels compared with placebo.
- Primary and secondary endpoints of cognition, whole brain blood flow, and cardiovascular function did not significantly improve over 12 weeks.
- Exploratory analyses revealed improved perfusion within the left hippocampus.
- Findings support the need for a larger, longer Phase III trial to evaluate NR's potential to delay cognitive decline and confirm regional changes in brain blood flow.

## 1 BACKGROUND

Despite recent advances in amyloid‐clearing therapies for Alzheimer's disease (AD),^1^, ^2^, ^3^ there remains a critical need for alternative or adjunctive treatment strategies that address the multifactorial nature of the disease.^4^ Monoclonal antibodies have demonstrated efficacy in reducing amyloid burden; however, their impact on cognitive outcomes has been modest, and their use is limited by high costs, complex administration, and the potential for serious adverse effects.^5^ These risks are particularly pronounced in individuals with concomitant cerebrovascular disease, for whom such therapies may be contraindicated.^6^ Given the strong association between vascular risk factors, including cerebral hypoperfusion, and AD pathogenesis,^7^, ^8^, ^9^ there is an urgent need to develop safe, accessible interventions that target vascular health as a means of preventing or slowing neurodegeneration.

Nicotinamide riboside (NR) is a naturally occurring precursor to nicotinamide adenine dinucleotide (NAD^+^), a critical coenzyme for cellular energy metabolism, and a co‐substrate for enzymes involved in DNA repair and mitochondrial function.^10^ Declining NAD^+^ levels with age have been implicated in the pathogenesis of numerous age‐related conditions, including neurodegenerative diseases such as AD.^11^, ^12^ Importantly, NAD^+^ also plays a central role in maintaining vascular health. Preclinical studies have shown that NAD^+^ repletion improves endothelial function, enhances nitric oxide bioavailability, and reduces arterial stiffness,^13^, ^14^ key factors in preserving cerebrovascular function. We have previously shown that oral supplementation with NR is well‐tolerated, raises blood NAD^+^ bioavailability, and may be effective for improving vascular health in older adults.^15^ Given the well‐established link between vascular risk factors and cognitive decline, these findings suggest that NR may exert neuroprotective effects, in part by improving vascular function, offering a promising approach for the prevention of dementia due to AD or vascular pathologies.

Clinical research evaluating the efficacy of NR for improving vascular function and brain health in older adults remains limited.^16^ Currently, only two small clinical studies with NR have been conducted in adults with mild cognitive impairment (MCI),^17^, ^18^ both of which reported no effect on cognitive function despite one reporting a 2.5‐fold increase in blood NAD^+17^. Here, we conducted a randomized, double‐blind, placebo‐controlled pilot clinical trial to assess the initial efficacy of NR supplementation for improving memory and brain blood flow in older adults with amnestic MCI (aMCI), while also examining its overall safety, adherence, and tolerability. The aMCI subgroup was chosen due to its greater likelihood of progressing to dementia due to AD. The primary outcome was the change in cognitive function over 12 weeks while secondary outcomes included whole brain cerebral perfusion, blood pressure, and arterial stiffness, to gain insight into potential mechanisms of action. Exploratory analyses included regional changes in cerebral blood flow (CBF). The findings from this study aim to help guide a larger, multi‐center, phase‐III trial.

## 2 METHODS

### 2.1 Trial design

The study design was a 12‐week double‐blind, randomized, placebo‐controlled parallel‐arm clinical trial of NR (500 mg b.i.d.) in older adults with aMCI. The trial was registered on clinicaltrials.gov on March 22, 2018, under the identifier NCT03482167. Participants were screened for inclusion between March 20, 2019, and August 21, 2023. All participants provided written informed consent prior to participation. The study protocol was approved by the University of Delaware Institutional Review Board (IRB#1079271) and adhered to the principles outlined in the Declaration of Helsinki. The complete protocol is available on clinicaltrials.gov. The trial was ended once enough participants had been enrolled to meet our primary objective with approval from the Data and Safety Monitoring Board (DSMB).

**RESEARCH IN CONTEXT**

1. **Systematic review**: We reviewed literature on vascular dysfunction in mild cognitive impairment (MCI) and Alzheimer's disease and on nicotinamide riboside (NR) as a modulator of nicotinamide adenine dinucleotide (NAD^+^)metabolism and neurovascular function. At the time our trial was initiated, no prior studies of NR in MCI had been published; however, two subsequent phase II trials have examined the effects of NR in similar populations. Collectively, these emerging findings provide important feasibility and biological rationale for future translation of this compound for AD prevention.
2. **Interpretation**: NR was well tolerated and increased blood NAD^+^ levels twofold. There were no significant improvements in cognitive function, whole brain blood flow, or cardiovascular function. Exploratory analyses revealed modest regional increases in cerebral blood flow (CBF) which should be examined in future trials.
3. **Future directions**: Larger, longer‐duration trials are needed to determine whether NR can slow cognitive decline and to clarify which patient subgroups may benefit most.

### 2.2 Participants

Community‐dwelling older adults (aged ≥ 65 years) with aMCI were recruited through the Delaware Center for Cognitive Aging Research (DECCAR), as previously described.^19^, ^20^ Recruitment was conducted via targeted mailings and social media advertisements seeking individuals with self‐reported memory concerns. Participants who scored between 21 and 34 on the modified Telephone Interview for Cognitive Status (TICS‐m),^21^ or ≤10 on the delayed recall portion of the TICS‐m to prioritize those with memory deficits, were invited for in‐person follow‐up screening. This screening assessment included the Mini‐Mental State Examination, 2^nd^ Edition (MMSE‐2),^22^ the study partner portion of the Clinical Dementia Rating (CDR), the Hopkins Verbal Learning Test‐Revised (HVLT‐R),^23^ and either the Brief Visuospatial Memory Test‐Revised (BVMT‐R)^24^ or the Logical Memory subtest (story A) from the Wechsler Memory Scale, 4th Edition (WMS‐IV). The BVMT‐R was administered in person until March 2020, when the study was temporarily paused due to the coronavirus disease 2019 (COVID‐19) pandemic. Subsequently, the WMS Logical Memory subtest was adopted for aMCI screening, as it could be administered remotely. Classification of aMCI was based on the clinical guidelines from the 2011 National Institute on Aging and the Alzheimer's Association workgroup^25^ and the Petersen criteria,^26^ which require (1) a self‐reported memory complaint, (2) objective memory performance ≥1.5 standard deviations below age‐adjusted norms, and (3) absence of dementia, indicated by a CDR global score < 1.0.

Participants were excluded if they had abnormal liver enzymes or renal function (estimated glomerular filtration rate < 30 mL per minute per 1.73 m^2^), any current systemic illness other than diabetes. Upon recommendation of the DSMB, we revised the protocol after study initiation to also exclude participants with any prior a history of cancer other than basal cell carcinoma. Additional exclusion criteria included major psychiatric disorders (e.g., schizophrenia, bipolar disorder, or major depressive disorder within the past 2 years), neurologic or autoimmune conditions affecting cognition (e.g., Parkinson's disease, epilepsy, multiple sclerosis, traumatic brain injury, or large‐vessel infarct), a concussion within the past 2 years, or a history of three or more concussions. Individuals were also excluded for substance use disorders, use of medications known to affect cognition (e.g., anticholinergics, or long‐acting benzodiazepines), current smoking (including marijuana) within the past 3 months, prior hospitalization for COVID‐19, or a history of cardiac arrhythmias or palpitations. Participants with contraindications to magnetic resonance imaging (MRI; e.g., claustrophobia, metal implants, or pacemakers) did not complete the MRI scan but were allowed to complete the remaining study visits.

### 2.3 AD biomarkers

The study protocol was written prior to publication of the revised criteria for the diagnosis and staging of AD^27^ and the emergence of blood biomarkers for AD pathology. Confirmation of AD pathology was not feasible at the time of study initiation due to the lack of positron emission tomography (PET) imaging capabilities at our center. However, considering current guidelines, we retrospectively measured plasma biomarkers of AD pathology to more completely characterize our cohort at baseline. We assessed plasma pTau217 based on its strong concordance with amyloid PET imaging^28^ and ability to discriminate AD from other neurodegenerative disorders.^29^ In addition, we measured amyloid‐β 42/40 ratio (Aβ42/40), neurofilament light chain (NfL), and glial fibrillary acidic protein (GFAP) using the Quanterix Neurology 4‐Plex E assay and white matter hyperintensity (WMH) volume to provide additional evidence of neurodegenerative or glial pathology. Blood biomarkers were assessed using a Quanterix SR‐X Biomarker Detection System using standardized protocols. WMH volume was measured from T2‐weighted images acquired on a Siemens Prisma 3T MRI scanner using Freesurfer software and the segmentation method described by Laso et al.^30^

### 2.4 Intervention

NR and matching placebo capsules were provided by Niagen Bioscience (formerly ChromaDex, Inc., Los Angeles, CA) through the ChromaDex External Research Program under a Material Transfer Agreement. Each active capsule contained 250 mg of NR, while placebo capsules were filled with microcrystalline cellulose and matched in size and color to ensure blinding. Participants were provided with a 12‐week supply of study capsules at baseline and instructed to take two capsules in the morning and two in the evening, for a total daily dose of 1000 mg of NR or placebo. The dose was selected based on our prior research demonstrating its safety/tolerability and efficacy for increasing blood NAD^+^ bioavailability and potentially lowering blood pressure and arterial stiffness.^15^ This dosage has also been evaluated in multiple clinical trials across a range of disease indications including older adults with MCI.^17^, ^31^, ^32^, ^33^, ^34^ The study was conducted under an Investigational New Drug (IND) application submitted to the United States Food and Drug Administration (FDA).

### 2.5 Randomization and blinding

Prior to group assignment, an unaffiliated data analyst from the UD Biostatistics Core generated a randomization scheme stratified by sex and apolipoprotein E (APOE) genotype, the strongest genetic risk factor for sporadic AD. The randomization sequence was generated using the built‐in randomization module in the electronic data capture system (REDCap). All study coordinators were blinded to actual treatment assignment but had access to blinded group assignment (A or B) to facilitate preparation of study capsules. All other investigators, including the lead biostatistician, remained fully blinded until after study completion and analysis of primary and secondary outcomes. The blinded key was securely stored by an unaffiliated faculty member in the PI's department and verified at the end of the study against lot numbers provided by the manufacturer.

### 2.6 Adherence, safety, and tolerability

Participants were instructed to return all capsules at the end of the study, and any unused capsules were counted to assess adherence. Safety and tolerability were monitored by the study coordinator during bi‐weekly check‐in visits, during which participants completed a structured symptom questionnaire by body system and an unstructured discussion about side effects. Standard blood chemistries were assessed at baseline and at the end of the intervention and any clinically abnormal values were reviewed by an independent DSMB appointed by the National Institute on Aging. All treatment‐emergent adverse events were documented according to their severity and potential relationship to the intervention and reviewed by the DSMB every six months.

### 2.7 NAD^+^ bioavailability

To confirm an increase in NAD^+^ bioavailability with NR treatment, whole blood was prepared using the method described by Demarest et al. with minor modifications (see Supplemental Methods for more detail).^35^ Participants were instructed not to take NR on the morning of testing to avoid any acute effects. Briefly, 20 µL of freshly drawn blood was pipetted into a 1.5 mL microfuge tube containing isotopically labeled internal standards. 120 µL of buffered ethanol solution was added to the sample and placed into a heat block at 80°C for 3 min. The sample was then centrifuged and 100 µL of the clear supernatant was transferred to an autosampler vial for analysis. Samples were stored at −80°C until being shipped on dry ice to the mass spectrometry laboratory for analysis. Chromatographic separation of the NAD^+^ metabolites was performed on an Agilent 1260 HPLC using a method described by Hsiao et al with minor modifications.^36^ Data for NAD^+^ metabolites was acquired on an Agilent 6490 triple quadrupole mass spectrometer in MRM mode using experimentally optimized conditions obtained by flow injection analysis of authentic standards for the following metabolites: nicotinamide adenine dinucleotide (NAD^+^, NADH), nicotinamide adenine dinucleotide phosphate (NADP^+^, NADPH), nicotinic acid adenine dinucleotide (NAAD), nicotinamide (NAM), NR, and nicotinamide mononucleotide (NMN). The acquired data were analyzed using Agilent Masshunter Quantitative Analysis software. We were unable to detect a signal for NR and NAAD in at least 50% of samples; therefore, these metabolites were excluded from the final analyses.

### 2.8 Primary outcomes

#### 2.8.1 Cognitive function

The primary outcome was the change in cognitive function at week 12 compared to baseline. Cognitive function was evaluated using raw scores from the California Verbal Learning Test Third Edition (CVLT‐III),^37^, ^38^ raw scores from the Wechsler Memory Scale (WMS‐IV) Logical Memory and Visual Reproduction subtests,^39^ and the Fluid Cognition Composite Score from the National Institutes of Health (NIH) Toolbox v2.0 Cognitive Battery.^40^ The CVLT‐III assesses verbal learning and memory by measuring one's ability to recall and recognize a list of words learned over five consecutive learning trials and again after short and long delays. The WMS‐IV subtests provide immediate and delayed recall scores from both story‐based (Logical Memory) and visual memory (Visual Reproduction) tasks. Stories B and C of the Logical Memory subtest were used for testing to avoid practice effects from Story A, which was used during the screening for aMCI. The NIH Toolbox v2.0 Cognition Battery evaluates multiple cognitive domains, including episodic memory, attention, working memory, processing speed, and executive function (inhibition and switching).

### 2.9 Secondary and exploratory outcomes

#### 2.9.1 CBF

Total CBF was assessed by pseudo‐continuous arterial spin labeling (pCASL) with multiple post‐labeling delays (PLDs) on a 3T Siemens Prisma MRI scanner with a 64‐channel head coil (Siemens, Erlangen, Germany). The pCASL protocol has been used previously by our group^41^ and was developed by the Human Connectome Project.^42^ Imaging parameters included: repetition time (TR) = 3705 ms; echo time (TE) = 26.4 ms; voxel size = 2.5 × 2.5 × 2.3 mm^3^; 90° flip angle, 215 × 215 mm^2^ field of view (FOV); 86 × 86 matrix, 43 slices, slice thickness 2.3 mm; 43 pairs of control/label images; total acquisition time = 5.5 min. The labeling duration was 1500 ms with five PLDs = 200 ms (control/label pairs = 6), 700 ms (pairs = 6), 1200 ms (pairs = 6), 1700 ms (pairs = 10), and 2200 ms (pairs = 15). Signal readout was implemented using 2D multi‐band gradient‐echo echo planar imaging (EPI) using phase partial Fourier = 6/8 and simultaneous multi‐slice (SMS) acceleration factor = 1. Brain blood flow is measured in milliliters of blood per minute per 100 g of brain tissue. High‐spatial‐resolution volumetric T1‐weighted magnetization‐prepared rapid acquisition gradient‐echo (MPRAGE) anatomical images of the brain were acquired for localization of brain structures with the following parameters: TR = 2300 ms, TE = 2.32 ms, inversion time (TI) = 900 ms; voxel size = 0.9 × 0.9 × 0.9 mm^3^ in‐plane spatial resolution; 8° flip angle; 256 × 256 matrix; 192 slices in a 3D acquisition. Total CBF was our pre‐specified secondary outcome; however, regional changes in CBF across the default mode network (DMN) were also included as exploratory outcomes to enable direct comparison with a similar trial by Orr et al.^17^ published after the start of our trial.^17^ We initially sought to also measure cerebrovascular reactivity to hypercapnia using transcranial Doppler ultrasound. However, we were unable to locate a suitable recording of the middle cerebral artery in most participants. These results have been reported on clinicaltrials.gov, although we are careful not to over interpret these findings.

#### 2.9.2 Cardiovascular function

Pre‐specified cardiovascular outcomes including seated blood pressure, aortic stiffness, and carotid artery compliance. Resting blood pressure was measured from the brachial artery in the seated position after at least 10 minutes of quiet rest using a semi‐automated oscillometric device (SunTech AdView 2, SunTech Medical Inc.). Repeated measurements were taken from the non‐dominant arm at 2‐minute intervals until three values within 5 mmHg were obtained and averaged. Aortic stiffness was assessed using carotid‐to‐femoral pulse wave velocity (PWV), the clinical gold‐standard measure of arterial stiffness.^43^ Measurement were obtained using the SphygmoCor XCEL system (AtCor Medical, Sydney, Australia), which automatically calculated PWV based on simultaneous recordings of carotid and femoral pressure waveforms.^43^, ^44^ Carotid artery compliance was measured using high‐resolution ultrasonography (LOGIQ e, GE Healthcare) to assess diameter changes in the right common carotid artery, and carotid pressure waveforms were obtained using the SphygmoCor CVMS system (AtCor Medical, Sydney, Australia), normalized to brachial pressure.^45^ Compliance was analyzed using Quipu software (Quipu srl, Pisa, Italy), as previously described,^46^ and calculated as CC = π × DD^2^ × (ΔD/DD) / (2 × PP), where DD is diastolic diameter, ΔD is diameter change, and PP is pulse pressure. Exploratory measures that indirectly assess arterial stiffness and wave reflection were obtained by radial artery pulse wave analysis (PWA), from which aortic pulse pressure and augmentation index (AIx) were derived using a validated transfer function.^43^

### 2.10 Sample size and statistical methods

Sample size estimates were based on effect sizes from our previous pilot study,^15^ targeting detection of improvements in blood pressure or arterial stiffness, which we hypothesized to be a key driver of cognitive benefits. Initial calculations indicated that 33 participants would provide ≥ 80% power, while 55 participants would exceed 95% power. Primary and secondary outcomes were analyzed by a biostatistician using a generalized linear mixed effects model from which we interpreted the treatment‐by time‐interaction. Effect size estimates were made based on a generalized *f* ^2^ for the fixed effect components of the mixed model and then converted to an equivalent Cohen's *d*. Two‐sided hypothesis testing was used for all analyses with the alpha (⍺) at ≤ 0.05. We did not correct for multiple comparisons to avoid an overly conservative design during this initial pilot phase. Due to the relatively low sample size in this phase‐II pilot study, we did not base our power calculation on seeing definitive effects on clinical outcomes and therefore did not include any covariates in the statistical model to avoid overinterpretation of our findings. Statistical analyses were limited to those who completed the study protocol using all available data. Sample sizes vary across outcomes because some participants had incomplete measures. The exact number of participants contributing to each analysis is reported in the *Supplemental Methods* (Table M1).

## 3 RESULTS

### 3.1 Participants

**FIGURE 1:**
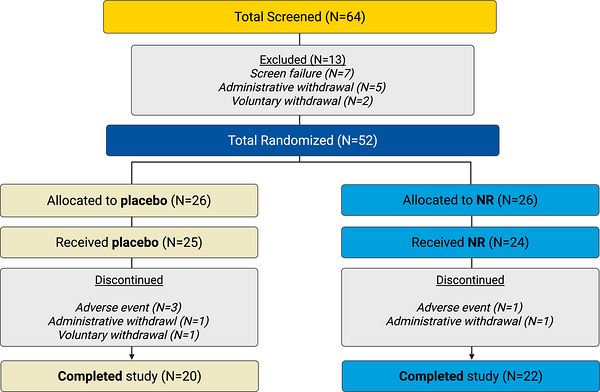
Participant flow diagram.

We screened 64 older adults (42F/22 M) with aMCI for inclusion in this study, reaching 87% of our prespecified enrollment target. Among those consented, 52 participants (33F/19 M) were randomized to treatment, and 42 participants (26F/16 M) completed the intervention, achieving 72% of our completion target (Figure 1). Three participants were randomized to treatment but were administratively withdrawn prior to receiving the intervention for starting new medications (*n* = 1) or disclosing a prior cancer diagnosis (*n* = 2). Among those randomized, 20 participants randomized to placebo completed the intervention, and 22 participants randomized to NR completed the intervention. A complete list of pre‐ and post‐randomization withdrawals are included in Tables S1 and S2, respectively. Participants were generally well‐matched between treatment groups by age, sex, APOE status, race and ethnicity, and baseline cognitive function (Table 1). However, the study included more women than men and there was a higher proportion of Black individuals in the placebo group compared to the NR group.

**TABLE 1:** Baseline characteristics of all participants that started treatment.

Variable	Placebo (*n* = 25)	NR (*n* = 24)	Total (*n* = 49)
Age—years	71 ± 7	72 ± 8	72 ± 8
Female sex—no. (%)	18 (72)	13 (54)	31 (63)
Male sex—no. (%)	7 (28)	11 (46)	18 (37)
Ethnicity—no. (%)			
Hispanic or Latino	1 (4)	1 (4)	2 (4)
Not Hispanic or Latino	24 (96)	23 (96)	47 (96)
Unknown or not reported	0 (0)	0 (0)	0 (0)
Race—no. (%)			
Asian	2 (8)	0 (0)	2 (4)
Black or African American	7 (28)	1 (4)	8 (16)
White	16 (64)	22 (92)	38 (78)
More than one race	0 (0)	1 (4)	1 (2)
APOE ε4 carrier—no. (%)	10 (40)	10 (42)	20 (41)
APOE genotype—no. (%)			
ε2/ε2	0 (0)	0 (0)	0 (0)
ε2/ε3	6 (24)	4 (17)	10 (20)
ε2/ε4	2 (8)	0 (0)	2 (4)
ε3/ε3	9 (36)	10 (42)	21 (39)
ε3/ε4	8 (32)	9 (38)	18 (35)
ε4/ε4	0 (0)	1 (4)	1 (2)
Cognitive function			
MMSE (raw score)	27 ± 2	26 ± 2	27 ± 2
CDR (global score)	0.4 ± 0.2	0.4 ± 0.2	0.4 ± 0.2
HVLT (T‐score)	39 ± 8	38 ± 8	39 ± 8
BVMT (T‐score)	35 ± 13; *n* = 4	33 ± 7; *n* = 8	35 ± 8; *n* = 12
WMS logical memory delayed recall (Scaled score)	8 ± 3; *n* = 21	8 ± 3; *n* = 16	8 ± 3; *n* = 37
Alzheimer's disease biomarkers			
Right hippocampal volume (mm^3^)	3597 ± 131; *n* = 17	3823 ± 110; *n* = 19	3716 ± 86; *n* = 36
Left hippocampal volume (mm^3^)	3467 ± 109; *n* = 17	3674 ± 106; *n* = 19	3576 ± 77; *n* = 36
White matter hyperintensity volume (cm^3^)	9.7 ± 1.5; *n* = 19	11 ± 1.6; *n* = 20	10 ± 1.1; *n* = 39
Plasma AD biomarkers	(*n* = 18)	(*n* = 19)	(*n* = 37)
pTau‐217 (pg mL^−1^)	0.38 ± 0.066	0.33 ± 0.049	0.35 ± 0.040
pTau‐217 positivity—no. (%)	7 (39)	5 (26)	12 (32)
Aβ_42/40_ (pg mL^−1^)	0.060 ± 0.003	0.066 ± 0.005	0.063 ± 0.003
NfL (pg mL^−1^)	21.2 ± 1.8	20.9 ± 1.9	21.1 ± 1.3
GFAP (pg mL^−1^)	167 ± 21	170 ± 15	169 ± 13

*Note*: Data are mean ± SEM. Race or ethnicity were reported by the participant. Screening for aMCI occurred in‐person until March 2020 when the study was temporarily stopped due to the COVID‐19 pandemic. Prior to the pandemic, we used the BVMT, in‐person, in our screening pipeline. After the pandemic, we began doing most of our screenings using online video conferencing. Because the BVMT cannot easily be administered online, we switched to using the WMS Logical Memory Test.

Abbreviations: APOE; apolipoprotein E gene; Aβ, amyloid‐β; BVMT, Brief Visuospatial Memory Test; CDR, Clinical Dementia Rating (informant interview only); GFAP, glial fibrillary acidic protein; HVLT, Hopkins Verbal Learning Test; MMSE, Mini‐Mental State Exmaination; NfL, neurofilament light; pTau, phosphorylated Tau; WMS, Wechsler Memory Scale.

### 3.2 AD biomarkers

Baseline plasma AD biomarkers for each treatment group and the entire cohort are summarized in Table 1. The mean pTau217 concentration was 0.35 ± 0.066 pg/mL with 12 participants (32%) exceeding the single‐point cutoff for Aβ positivity (> 0.42 pg/mL) reported by Ashton et al.^28^ These data indicate AD pathology in about 30% of participants, which is consistent with other reports of community‐dwelling adults with aMCI.^47^ While standardized cutoffs for other biomarkers are not yet established, a plasma Aβ42/40 ratio below 0.0465 has been proposed as a potential cutoff for Aβ positivity using the Simoa‐based neurology three‐plex assay.^48^ In our cohort, the mean Aβ42/40 (using the Simoa NP4E assay) was 0.063 ± 0.003 [range: 0.030–0.122] with 21 participants below the proposed cutoff. Additionally, plasma NfL values were moderately elevated compared to published reference ranges for adults in this age range (9.1–47.1 pg/mL).^49^, ^50^ The mean GFAP concentration was near the upper end of a reference range for adults aged 60–79 years (40.7–227.5), which may indicate co‐occurrence of neuroinflammation or vascular pathology. The mean WMH volume (10 ± 1.1 cm^3^) was near the 75th percentile this age group,^51^ suggesting moderate burden of small vessel disease.

### 3.3 Adherence, safety, and tolerability

Adherence was similar between groups with 85 ± 5% adherence in the NR group and 91 ± 1% adherence in the placebo group (*p* = 0.22). Clinical laboratory values remained stable; only ALT, relative monocytes, and mean corpuscular volume (MCV) showed statistically significant group‐by‐time interactions, though these changes were small not clinically meaningful (Tables S3–S5). No serious adverse events were reported in either the NR or placebo groups (Table S6). The most frequently reported adverse events in the NR group included gastrointestinal symptoms such as nausea and diarrhea, as well as mild headaches. These events were transient and did not necessitate discontinuation of the intervention. The placebo group experienced similar types and frequencies of adverse events. Four participants discontinued treatment due to adverse events, including three randomized to placebo and one randomized to NR. The reasons for these withdrawals are described in Table S2 and include complaints of flatulence and sleep disturbance, heart palpitations, and chest pain in the placebo group and low laboratory measurement of estimated glomerular filtration rate (eGFR) based on serum creatinine in the NR group. Overall, the incidence of adverse events did not differ significantly between the NR and placebo groups, indicating that NR supplementation was well tolerated.

### 3.4 NAD^+^ bioavailability

Consistent with prior studies of NR supplementation reporting an increase in NAD^+^ metabolites or catabolites,^15^, ^17^, ^31^, ^33^, ^52^ we observed a robust increase in blood NAD^+^ concentration in the NR group compared to placebo (Table 2), with levels nearly doubling from baseline. Only two participants in the NR group exhibited either no change or a decrease blood NAD^+^ concentration, further supporting our conclusion of strong compliance to NR in this study. No change in NAD^+^ was observed in the placebo group (Table 2). A similar pattern was seen for NADH, which increased significantly in the NR group but remained unchanged in the placebo group. Although other NAD‐related metabolites did not change significantly from baseline, most were elevated during NR supplementation, contributing to a significant overall increase in the total NAD metabolome, primarily driven by changes in NAD^+^ and NADH.

**TABLE 2:** NAD^+^ metabolites.

	Placebo (*N* = 17)		NR (*N* = 19)		Tx*time	Effect size
Parameter	Baseline	Week 12	Baseline	Week 12	*p*‐Value	*d*
Total NADome	44.35 ± 3.24	45.61 ± 2.55	41.84 ± 3.31	70.71 ± 5.07	<0.001	1.499
NAD^+^	24.12 ± 1.70	25.14 ± 1.58	23.36 ± 1.94	48.52 ± 4.12	<0.001	1.879
NADH	0.37 ± 0.07	0.43 ± 0.11	0.36 ± 0.10	0.79 ± 0.19	0.022	0.795
NADP	12.24 ± 1.35	12.68 ± 0.98	10.66 ± 0.83	11.41 ± 0.68	0.830	0.073
NADPH	2.50 ± 0.70	1.70 ± 0.42	1.78 ± 0.47	1.92 ± 0.46	0.188	0.449
NaM	3.44 ± 0.50	4.27 ± 0.66	4.19 ± 0.90	6.23 ± 0.89	0.358	0.313
NMN	1.67 ± 0.12	1.60 ± 0.12	1.70 ± 0.19	1.79 ± 0.16	0.292	0.346

*Note*: Data are estimated means ± SE. Units in µM. Effect size estimates were made based on a generalized *f* ^2^ for the fixed effect components of the mixed model and then converted to an equivalent Cohen's *d*. *p*‐Values are not corrected for multiple comparisons. Total NADome reflects the sum of all NAD‐related metabolites.

Abbreviations: NAD/NADH, nicotinamide adenine dinucleotide; NADP/NADPH, nicotinamide adenine dinucleotide phosphate; NaM, nicotinamide; NaM, nicotinamide; NMN, nicotinamide mononucleotide; NR, nicotinamide riboside; Tx, treatment.

### 3.5 Primary outcomes

#### 3.5.1 Cognitive function

Both groups exhibited modest within‐group increases in cognitive function indicating possible practice effects. However, there were no group‐by‐time interactions indicating no significant improvement with NR over the 12‐week intervention period (Table 3). The delayed recall component of the Logical Memory subtest exhibited the largest effect size (*d* = 0.607), although the interaction was not statistically significant (*p* = 0.062).

**TABLE 3:** Cognitive function.

	Placebo (*N* = 20)		NR (*N* = 22)		Tx*time	Effect size
Parameter	Baseline	Week 12	Baseline	Week 12	*p*‐Value	*d*
**CVLT‐III—raw scores**						
Trial 1–5	39 ± 3	42 ± 2	40 ± 3	41 ± 4	0.426	0.2091
Delayed RECALL	7 ± 1	8 ± 1	7 ± 1	9 ± 1	0.836	0.066
Discriminability score	83 ± 3	82 ± 3	83 ± 3	84 ± 3	0.562	0.186
**WMS‐IV—raw scores**						
Logical memory I	18 ± 1	21 ± 1	20 ± 2	25 ± 2	0.322	0.319
Logical memory II	15 ± 1	16 ± 2	15 ± 2	19 ± 2	0.062	0.607
Logical recognition	23 ± 1	23 ± 1	22 ± 1	24 ± 1	0.243	0.376
Visual reproduction I	29 ± 1	30 ± 2	29 ± 2	30 ± 2	0.850	0.061
Visual reproduction II	17 ± 2	19 ± 2	17 ± 2	18 ± 2	0.396	0.273
Visual recognition	5 ± 0.4	6 ± 0.3	5 ± 0.3	5 ± 0.4	0.118	0.513
**NIH toolbox**						
Fluid cognition score	87 ± 2	89 ± 2	84 ± 3	84 ± 3	0.398	0.272

*Note*: Data are estimated means ± SE. Effect size estimates were made based on a generalized *f* ^2^ for the fixed effect components of the mixed model and then converted to an equivalent Cohen's *d*. *p*‐values are not corrected for multiple comparisons.

Abbreviations: NR, nicotinamide riboside; Tx, treatment, CVLT‐III, California Verbal Learning Test III; WMS‐IV, Weschler Memory Scale IV.

### 3.6 Secondary and exploratory outcomes

#### 3.6.1 CBF

Total CBF (secondary endpoint) was not significantly improved in the NR group relative to placebo. However, we observed a mean change of 7.2 mL/min/100 g relative to baseline in the NR group that was not evident in the placebo group (*p* = 0.135). To compare our findings with Orr et al.,^17^ which was published after the initiation of this trial, we conducted an exploratory analysis of regional changes in CBF across the DMN.^17^ Several subregions showed promising trends towards improvement with NR with moderately large effect sizes (Table 4). Most of these changes did not reach statistical significance except for the left hippocampus, and none would have survived correction for multiple comparisons.

**TABLE 4:** Cerebral blood flow.

	Placebo (*N* = 17)		NR (*N* = 19)		Tx*time	Effect size
Parameter	Baseline	Week 12	Baseline	Week 12	*p*‐Value	*d*
Total CBF	47.2 ± 2.8	47.2 ± 2.6	48.5 ± 4.1	55.7 ± 3.8	0.135	0.512
White matter	32.6 ± 2.8	32.0 ± 1.7	33.0 ± 2.3	38.1 ± 2.7	0.140	0.505
Total DMN	44.0 ± 2.6	43.3 ± 2.5	43.5 ± 3.8	51.7 ± 3.7	0.079	0.603
Precuneus	43.5 ± 3.4	42.8 ± 3.0	41.6 ± 4.0	50.6 ± 4.1	0.091	0.579
PCC	58.5 ± 3.7	54.7 ± 3.8	55.6 ± 4.7	64.6 ± 4.7	0.057	0.654
r. IPL	36.4 ± 2.7	36.7 ± 3.2	36.5 ± 4.1	44.6 ± 3.5	0.091	0.516
l. IPL	36.9 ± 3.2	36.4 ± 3.1	35.6 ± 3.6	47.3 ± 4.8	0.132	0.643
v. ACC	56.2 ± 3.3	54.8 ± 2.9	52.0 ± 4.2	59.6 ± 4.0	0.836	0.579
m. PFC	38.0 ± 3.1	37.6 ± 3.3	45.8 ± 5.0	44.4 ± 4.5	0.836	0.003
r. MTG	44.2 ± 2.8	43.0 ± 3.2	45.4 ± 4.0	52.0 ± 3.4	0.062	0.531
l. MTG	44.8 ± 2.7	43.1 ± 2.7	45.4 ± 3.8	53.7 ± 4.4	0.102	0.561
Total Hippo	54.9 ± 2.8	51.4 ± 2.1	51.2 ± 3.6	56.7 ± 3.8	0.050	0.676
r. Hippo	54.3 ± 3.1	51.2 ± 2.3	51.1 ± 3.9	55.4 ± 3.9	0.120	0.533
l. Hippo	55.6 ± 2.8	51.7 ± 2.3	51.7 ± 3.3	58.0 ± 3.8	0.033	0.736

*Note*: Data are estimated means ± SE in mL/min/100 g. Effect size estimates were made based on a generalized *f* ^2^ for the fixed effect components of the mixed model and then converted to an equivalent Cohen's *d*. *p*‐values are not corrected for multiple comparisons.

Abbreviations: CBF, cerebral blood flow; DMN, default mode network; Hippo, right and left hippocampus; IPL, right and left inferior parietal lobe; m. PFC, medial prefrontal cortex; NR, nicotinamide riboside; PCC, posterior cingulate cortex; right and left MTG, mid temporal gyrus; Tx, treatment; v. ACC, ventral anterior cingulate cortex.

#### 3.6.2 Cardiovascular function

NR supplementation was associated with modest reductions in blood pressure and arterial stiffness (secondary endpoints), although these changes were not statistically significant (Table 5). Carotid‐femoral PWV and carotid artery compliance did not change with NR supplementation; however, aortic AIx, an exploratory endpoint and indirect measure of arterial stiffness and wave reflection, decreased in the NR group and increased in the placebo group, yielding a moderately large effect size (*d* = 0.628; *p* = 0.054). These findings suggest that NR may exert favorable effects on vascular function, particularly in measures of blood pressure and wave reflection, though larger studies are needed to confirm these effects.

**TABLE 5:** Blood pressure and arterial stiffness

	Placebo (*N* = 20)		NR (*N* = 22)		Tx*Time	Effect size
Parameter	Baseline	Week 12	Baseline	Week 12	*p*‐Value	*d*
HR, bpm	63 ± 3	64 ± 2	64 ± 2	63 ± 2	0.195	0.418
SBP, mmHg	124 ± 3	123 ± 2	132 ± 3	127 ± 3	0.187	0.427
DBP, mmHg	70 ± 2	72 ± 2	74 ± 2	73 ± 2	0.052	0.631
PP, mmHg	54 ± 2	51 ± 2	58 ± 2	54 ± 3	0.696	0.126
PWV, cm/sec	8.9 ± 0.4	8.5 ± 0.5	9.8 ± 0.4	9.1 ± 0.5	0.552	0.196
Carotid compliance,	0.76 ± 0.09	0.60 ± 0.06	0.81 ± 0.09	0.69 ± 0.09	0.505	2.270
Aortic AIx, %	35 ± 2	38 ± 2	39 ± 2	34 ± 2	0.054	0.628
Aortic PP, mmHg	52 ± 3	53 ± 3	53 ± 3	54 ± 3	0.830	0.069

*Note*: Data are estimated means ± SE. Effect size estimates were made based on a generalized *f* ^2^ for the fixed effect components of the mixed model and then converted to an equivalent Cohen's *d*. *p*‐values are not corrected for multiple comparisons.

Abbreviations: AIx, augmentation index; DBP, diastolic blood pressure; HR, heart rate; NR, nicotinamide riboside; PP, pulse pressure; PWV, pulse wave velocity; SBP, systolic blood pressure; Tx, treatment.

## 4 DISCUSSION

In this randomized, placebo‐controlled pilot trial, 12 weeks of NR supplementation was well‐tolerated and significantly increased blood NAD^+^ concentration in older adults with aMCI. However, these effects were not accompanied by significant improvements in cognitive function (primary endpoint), total brain blood flow, or cardiovascular function (secondary endpoints). Exploratory analyses revealed small changes in CBF that are biologically plausible; however, these findings were not corrected for multiple comparisons and should be interpreted cautiously and confirmed in adequately powered future trials. Collectively, these phase‐II findings provide important preliminary evidence of feasibility to guide the design of future pivotal trials of NR for the treatment or prevention of AD.

We observed a two‐fold increase in blood NAD^+^ concentration, consistent with prior clinical studies of NR.^15^, ^53^, ^54^ This finding suggests good adherence to NR among older adults with MCI, which is an important consideration for translation in this population. We did not detect NR itself in whole blood; however, this is not surprising as orally administered NR is believed to be metabolized by gut bacteria to nicotinamide and nicotinic acid and subsequently converted to NAD^+^ in blood via the Preiss–Handler pathway.^55^, ^56^, ^57^ Whether the observed increase in blood NAD^+^ translated to an increase in brain NAD^+^ in this study is not known. Evidence from MR‐spectroscopy and neuron‐derived extracellular vesicles suggests oral NR can increase brain NAD^+^ in humans^53^, ^58^, ^59^; however, large interindividual variability and a delayed brain uptake may warrant a longer treatment duration and larger sample size to observe functional effects.

Despite not meeting prespecified primary and secondary end‐points, our exploratory analyses revealed possible effects on regional perfusion, particularly within the hippocampus. The hippocampus is among the earliest brain regions affected by AD^20^, ^60^, ^61^ and the left hippocampus is closely involved in the consolidation of delayed recall of verbal information.^62^ This is notable as logical memory exhibited the largest effect size with NR supplementation; however, these findings should be interpreted cautiously and regarded as hypothesis‐generating, as they were not corrected for multiple comparisons and the observed effects on cognitive function were not significant.

Our exploratory CBF findings conflict with those of Orr et al.,^17^ who reported a reduction in CBF within the DMN following NR supplementation in older adults with MCI.^17^ However, several key differences may explain these divergent results. Orr et al. used a dose‐escalation strategy over 10 weeks, gradually increasing NR to a final dose of 1000 mg/day, whereas our study administered 1000 mg/day (500 mg b.i.d.) throughout the 12‐week intervention. The immediate and sustained exposure to NR in our study may have produced more robust or sustained vascular effects. Participant demographics may have also influenced study outcomes as Orr et al. included predominantly Hispanic older adults, a group that typically exhibits a higher prevalence of cardiometabolic risk factors known to affect cerebrovascular health.^63^ In contrast, our sample included more white and non‐white Hispanics and exhibited only moderate cerebrovascular disease based on WMH volume, potentially allowing for greater improvements in cerebral perfusion. Thus, the overall responsiveness to NR may be limited in those with more severe cerebrovascular pathology, suggesting NR may be most appropriately administered for primary or secondary prevention of cerebrovascular diseases.

Our sample was too small to investigate meaningful predictors of responsiveness to NR; however, we noted that individuals with lower baseline CBF exhibited larger improvements with NR supplementation, suggesting potential greater efficacy among those with compromised cerebrovascular function. The exact mechanisms by which NR may increase CBF remain incompletely understood. Other NAD^+^ precursors have been shown to improve vascular endothelial function in the peripheral circulation by activating the sirtuin‐1 (SIRT1) and sirtuin‐3 (SIRT3) enzymes. These enzymes promote cellular stress resistance, reduced oxidative stress, and enhanced endothelial nitric oxide production. NAD^+^ is also essential for facilitating energy production and regulating mitochondrial biogenesis, both of which are necessary to support the high metabolic demands of the brain and contribute to appropriate neurovascular coupling. Whether our findings primarily reflect an improvement in cerebrovascular endothelial function, improved brain energy metabolism, or both remain to be determined; however, these mechanisms should be further investigated in future studies.

Contrary to our a priori hypothesis, NR was associated with only small improvements in blood pressure and arterial stiffness that were not statistically significant. However, the observed decrease in aortic AIx, a measure of arterial pressure wave reflection and surrogate marker of arterial stiffness, suggests potential engagement with peripheral vascular function that may not have been fully captured due to sample size limitations or treatment duration. Our earlier work suggests NR supplementation may significantly reduce systolic blood pressure and carotid–femoral PWV in older adults, particularly among individuals with elevated baseline blood pressure.^15^ One possible explanation for this discrepancy is that the current study did not specifically enroll participants based on their baseline blood pressure status. Baseline blood pressure was more variable and generally lower in the current study, which might have prevented the detection of statistically significant treatment effects. Future studies should consider stratifying participants by baseline blood pressure status or vascular health to better understand the conditions under which NR exerts its greatest physiological effects.

Several limitations should be considered when interpreting these findings. Participants were recruited solely based on a research diagnosis of aMCI using behavioral assessments, as this study was initiated prior to the widespread availability of reliable blood biomarkers for AD pathology.^64^ We retrospectively measured pTau‐217, as this biomarker has been shown to be highly correlated with PET and cerebrospinal fluid (CSF) measures of amyloid‐β^65^; however only one third of our participants met our defined biomarker criteria for AD‐related pathology. This is consistent with the prevalence of pTau‐217 positivity among community‐dwelling adults with MCI and it is possible that some of our participants had emerging AD pathology that did not yet meet criteria for amyloid positivity.^47^ This limitation underscores the importance of incorporating biomarker‐defined enrollment criteria in future trials as heterogeneity in pathology may have influenced the treatment response. While our sample size was sufficient to detect moderate effects on vascular function, we are likely underpowered to assess responsiveness to the intervention based on factors APOE genotype or baseline AD biomarker status. It is plausible that the effects of NR on cognitive outcomes differ depending on these factors. Future studies should incorporate biomarker‐based stratification during enrollment to better understand the interaction between NR supplementation and disease biology. We limited our analyses of these phase II data to those who completed the protocol; however future phase III trials should include an intention to treat analysis to evaluate the real world efficacy of NR in this population.

### 4.1 Summary and conclusion

In summary, 12 weeks of NR supplementation was well tolerated and effectively increased blood NAD^+^ concentration but did not significantly improve cognitive function, whole brain perfusion, or cardiovascular function in older adults with amnestic MCI. Exploratory analyses reveal more subtle effects on regional CBF that warrant follow‐up in future trials. The short duration of the intervention, heterogeneity of our sample with respect to AD pathology, and lack of control for other co‐variates may have contributed to the lack of robust efficacy in this trial. Future studies should incorporate biomarker‐based stratification and larger sample sizes to determine whether the effects of NR differ based on disease etiology and to confirm its potential as a therapeutic strategy for cognitive decline.

## CONFLICT OF INTEREST STATEMENT

The authors have no conflicts of interest to disclose. Author disclosures are available in the Supporting Information.

## CONSENT STATEMENT

All participants provided written informed consent prior to participation.

## Supporting information




Supporting Information

Supporting Information

Supporting Information
