# Supplementary material for: A phase‐II randomized controlled pilot study of nicotinamide riboside supplementation in older adults with amnestic mild cognitive impairment
Source: Alzheimers Dement. 2026 Jul 21;22(7):e71605. doi: 10.1002/alz.71605 (PMC13386481; doi:10.1002/alz.71605)
Supplement: Supplementary file 2 — Supporting Information [file ALZ-22-e71605-s003.docx]

| **Table M1.** List of Outcome Measures and Sample Size Analyzed | | | | | |
| --- | --- | --- | --- | --- | --- |
| **Outcome** | **Table** | **Placebo (N=20)** | | **NR (N=22)** | |
|  |  | **Baseline (n)** | **Week 12 (n)** | **Baseline (n)** | **Week 12 (n)** |
| NAD+ Metabolites |  |  |  |  |  |
| - All Measures | Table 2 | 17 |  | 18 | 20 |
| Cognitive Function |  |  |  |  |  |
| - CVLT-III (all measures) | Table 3 | 20 | 20 | 22 | 21 |
| - WMS-IV |  |  |  |  |  |
| - - Logical Memory I | Table 3 | 20 | 20 | 22 | 21 |
| - - Logical Memory II | Table 3 | 20 | 20 | 22 | 21 |
| - - Logical Recognition | Table 3 | 20 | 20 | 22 | 21 |
| - - Visual Reproduction I | Table 3 | 20 | 20 | 22 | 22 |
| - - Visual Reproduction II | Table 3 | 20 | 20 | 22 | 22 |
| - - Visual Recognition | Table 3 | 20 | 20 | 20 | 21 |
| - NIH Toolbox |  |  |  |  |  |
| - - Fluid Cognition Score | Table 3 | 20 | 20 | 21 | 22 |
| Cerebral Blood Flow |  |  |  |  |  |
| - All Measures | Table 4 | 17 | 17 | 19 | 18 |
| Blood Pressure and Arterial Stiffness |  |  |  |  |  |
| - Heart Rate | Table 5 | 20 | 20 | 22 | 22 |
| - Systolic BP | Table 5 | 20 | 20 | 22 | 22 |
| - Diastolic BP | Table 5 | 20 | 20 | 22 | 22 |
| - Pulse Pressure | Table 5 | 20 | 20 | 22 | 22 |
| - Pulse Wave Velocity | Table 5 | 19 | 18 | 21 | 21 |
| - Carotid Compliance | Table 5 | 20 | 19 | 17 | 16 |
| - Aortic AIx | Table 5 | 20 | 20 | 22 | 22 |
| - Aortic Pulse Pressure | Table 5 | 20 | 20 | 22 | 22 |
| CBC and Hematology |  |  |  |  |  |
| - Prothrombin (INR) | Table S3 | 19 | 15 | 20 | 20 |
| - Prothrombin Time | Table S3 | 19 | 15 | 20 | 20 |
| - Thromboplastic Time | Table S3 | 19 | 15 | 20 | 20 |
| - All other measures | Table S3 | 20 | 20 | 22 |  |
| Comprehensive Metabolic Panel |  |  |  |  |  |
| - All Measures | Table S4 | 20 | 20 | 22 | 22 |
| Total lipid panel |  |  |  |  |  |
| - VLDL | Table S5 | 20 | 20 | 21 | 21 |
| - All other Measures | Table S5 | 20 | 20 | 22 | 22 |

***Detailed Method for Quantifying NAD^+^ Bioavailability***

**Materials**. NAAD, NAD+, NADP+, NADH, NADPH, NaM, NmN, and adenosine triphosphate, cytidine monophosphate-13C9-15N3, 200 proof ethanol were obtained from Sigma Aldrich (St. Louis, MO). Sodium hydroxide and HEPES were obtained from Fisher Scientific (Fairlawn, NJ). Adenosine triphosphate (ribose-d4) and metabolite yeast extract (U13C, 98%, product #ISO1) were obtained from Cambridge Isotope Laboratories (Tewksbury, MA). NaM (13C6) was obtained from Cerilliant (Round Rock, TX). Nicotinamide riboside and doubly labeled nicotinamide riboside (13C1, H2-1) were obtained from Chromadex Inc. (Irvine, CA). All HPLC solvents and extraction solvents were HPLC grade or better.

**Preparation of calibration standards**. Individual stock standards for NAAD, NAD+, NADP+, NaM, NmN, and adenosine triphosphate were prepared by dissolving 10 mg/ml in deoxygenated buffered ethanol solution (3:1 ethanol:1mM HEPES pH 7.1) and then combining to obtain a stock mixture at various concentrations depending on the amount expected for each compound (mix A). The NADH and NADPH combined stock standard (mix B) was prepared separate from the other compounds. both were frozen at −70°C until use.

The internal standard solution was prepared at 250 μg/ml adenosine triphosphate-d4, doubly labeled nicotinamide riboside and 2.5 μg/ml of NaM-13C6 in deoxygenated buffered ethanol solution. The metabolite yeast extract containing the U13C labeled NAD+ and NADP+ was prepared by adding 2ml of deoxygenated buffered ethanol solution to 15mg of lyophilized yeast extract and vortexed until the extract was completely reconstituted. The reconstituted yeast extract was then centrifuged at 14,000 RPM for 5 minutes at 4°C, and the clear supernatant was then stored in 50μl aliquots in 1.5ml microfuge tubes at -70°C until use. Immediately prior to use, 250μl of deoxygenated buffered ethanol solution was added to the 50μl aliquot of yeast extract and vortexed for 10 seconds.

A calibration stock standard was prepared by adding 20μl of internal standard, 10μl of yeast extract, 15μl of mix A, 15μl of mix B and 90 μl of deoxygenated buffered ethanol solution (total volume=150μl). The calibration stock solution was then diluted into 9 additional calibration standards in deoxygenated buffered ethanol solution, with the internal standard and yeast extract concentration kept constant in the 10 calibration levels. The concentration of NAD+ in the 10 calibration standards was 30.2, 15.1, 7.55, 3.77, 1.88, 0.944, 0.472, 0.236, 0.0472 and 0.0236 μM respectively.

**Extraction of NAD+ metabolites from whole blood**. Whole blood was prepared using the method described by Demarest et al^1^. Briefly, 20ul of freshly drawn blood was pipetted into a 1.5ml microfuge tube and combined with 20μl of internal standard, 10μl of yeast extract and 120μl of deoxygenated buffered ethanol solution preheated to 80^°^C. The sample was then placed into a heat block at 80^°^C for 1.5 minutes. The sample was vortexed for 10 seconds and then placed back into the heat block for an additional 1.5 minutes. The sample was then centrifuged at 13,200xg for 10 minutes at 4°C. 100μl of the clear supernatant was then transferred to an autosampler vial for analysis. Samples were stored at -70°C until being shipped on dry ice to the mass spectrometry laboratory for analysis.

**LC-MS**. HPLC separation of NAD+ metabolites was performed using a method described by Hsiao et al with minor modifications^2^. Separation of NAD+ metabolites was performed on a 1200 series HPLC with a 150 × 2.1 mm Poroshell 120 HILIC-Z column from Agilent Technologies (Santa Clara, CA). Buffer A consisted of 10mM ammonium acetate adjusted to pH 9.0 with ammonium hydroxide, and buffer B consisted of 90:10 acetonitrile:water with 10mM ammonium acetate adjusted to pH 9.0 with ammonium hydroxide. 5uM of methylenediphosphonic acid was added to both buffers. Two microliters of the extracted sample was analyzed using the following gradient at a flow rate of 0.5 ml/min: Hold at 90% B for 2 min, then 90% B to 60% B from 2 to 12 min, Hold at 60% B from 12 to 15 min, then 60% B to 90% B from 15 to 6 minutes followed by reequilibration at 90% B for 5 minutes. The column temperature was held at 25°C for the entire gradient. Mass spectrometric analysis was performed on an Agilent 6490 triple quadrupole mass spectrometer with an electrospray source in positive ionization mode. The drying gas was 130°C at a flow rate of 15L/min. The nebulizer pressure was 35psi. The sheath gas temperature was 350°C at a flow rate of 12L/min. The capillary voltage was 3000V. Data for NAD+ metabolites was acquired in MRM mode using experimentally optimized conditions obtained by flow injection analysis of authentic standards.

**Data Analysis**. Calibration curves for each NAD+ metabolite were constructed using Agilent Masshunter Quantitative Analysis software. The peak areas for U13C-NAD+, U13C-NADP+ and U13C-NADH from the yeast extract were used as internal standards for their corresponding unlabeled metabolites. The peak area for U13C-NADP+ in the yeast extract was used as the internal standard for unlabeled NADPH. Results were quantitated using these calibration curves to obtain the concentration of the NAD+ metabolites in μM of each metabolite in whole blood.

1 Demarest, T. G. *et al.* Assessment of NAD(+)metabolism in human cell cultures, erythrocytes, cerebrospinal fluid and primate skeletal muscle. *Anal Biochem* **572**, 1-8, doi:10.1016/j.ab.2019.02.019 (2019).

2 Hsiao, J. J., Potter, O. G., Chu, T. W. & Yin, H. Improved LC/MS Methods for the Analysis of Metal-Sensitive Analytes Using Medronic Acid as a Mobile Phase Additive. *Anal Chem* **90**, 9457-9464, doi:10.1021/acs.analchem.8b02100 (2018).
