# Supplementary material for: A phase‐II randomized controlled pilot study of nicotinamide riboside supplementation in older adults with amnestic mild cognitive impairment
Source: Alzheimers Dement. 2026 Jul 21;22(7):e71605. doi: 10.1002/alz.71605 (PMC13386481; doi:10.1002/alz.71605)
Supplement: Supplementary file 3 — Supporting Information [file ALZ-22-e71605-s002.docx]

| **Table S1.** Reasons for withdrawal prior to randomization | |
| --- | --- |
| Participant | Reason (N=13) |
| Screen Failure | N = 7 |
| NAD003 | History of concussion |
| NAD042 | History of cancer |
| NAD043 | History of cancer |
| NAD044 | History of cancer |
| NAD046 | History of non-malignant mass |
| NAD048 | Major depressive disorder |
| NAD050 | History of cardiac arrythmia |
| Administratively Withdrawn | N = 5 |
| NAD014 | Lost to follow-up |
| NAD025 | Lost to follow-up |
| NAD032 | Failure to complete study protocol |
| Voluntary Withdrawal | N = 2 |
| NAD008 | Concerns regarding COVID-19 |
| NAD022 | Lack of time commitment |

| **Table S2.** Reasons for withdrawal after randomization | | | |
| --- | --- | --- | --- |
| Randomized to NR | | | |
| Participant | Type | Study Timepoint | Reason |
| NAD006 | Administrative | Week 4 | Prescribed new medication (anticholinergic) |
| NAD034 | Administrative | Week 0 | Disclosed prior cancer history (before starting study pills) |
| NAD051 | Adverse Event | Week 6 | Low eGFR |
| NAD055* | Administrative | Week 0 | Disclosed prior cancer history (before starting study pills) |
| Total Withdrawn | | | n = 4 out of 26 randomized (15%) |
| Randomized to Placebo | | | |
| Participant | Type | Study Timepoint | Reason |
| NAD012 | Administrative | Week 2 | Prescribed new medication (angiotensin receptor antagonist) |
| NAD036 | Voluntary | Week 2 | Time constraints |
| NAD040 | Adverse Event | Week 1 | Complaint of flatulence and sleep disturbance |
| NAD045 | Adverse Event | Week 4 | Heart palpitations |
| NAD054 | Adverse Event | Week 2 | Chest pain |
| NAD056 | Administrative | Week 0 | Disclosed prior cancer history (before starting study pills) |
| Total Withdrawn | | | n = 6 out of 26 randomized (23%) |

| **Table S3.** Complete blood count and hematology | | | | | |
| --- | --- | --- | --- | --- | --- |
|  | **Placebo** | | **NR** | | **Tx*Time** |
|  | **Baseline** | **Week 12** | **Baseline** | **Week 12** | **p-value** |
| WBC (10^6^ uL^-1^) | 6.0 ± 0.4 | 5.9 ± 0.3 | 6.1 ± 0.2 | 6.2 ± 0.3 | 0.655 |
| RBC (10^6^ uL^-1^) | 4.5 ± 0.1 | 4.5 ± 0.1 | 4.5 ± 0.1 | 4.5 ± 0.1 | 0.569 |
| HGB (g dL^-1^) | 13.8 ± 0.3 | 12.9 ± 0.3 | 13.9 ± 0.4 | 13.8 ± 0.3 | 0.709 |
| HCT (%) | 42 ± 1 | 42 ± 1 | 41 ± 1 | 41 ± 1 | 0.889 |
| MCV (fL) | 93 ± 1 | 92 ± 1 | 91 ± 1 | 92 ± 1 | 0.023 |
| MCH (pg) | 31 ± 0.4 | 31 ± 0.4 | 31 ± 0.4 | 31 ± 0.4 | 0.664 |
| MCHC (g dL^-1^) | 33 ± 0.2 | 33 ± 0.2 | 34 ± 0.2 | 33 ± 0.2 | 0.282 |
| RDW (%) | 12.8 ± 0.2 | 12.9 ± 0.2 | 12.9 ± 0.1 | 13.0 ± 0.2 | 0.711 |
| Platelet count (10^3^ uL^-1^) | 252 ± 18 | 240 ± 15 | 229 ± 10 | 229 ± 9 | 0.290 |
| Prothrombin (INR) | 1.0 ± 0.01 | 1.1 ± 0.03 | 1.0 ± 0.02 | 1.1 ± 0.02 | 0.291 |
| Prothrombin Time (sec) | 10.9 ± 0.1 | 11.4 ± 0.3 | 10.9 ± 0.1 | 11.3 ± 0.2 | 0.723 |
| Thromboplastin Time (sec) | 28.7 ± 0.5 | 29.5 ± 0.8 | 28.6 ± 0.4 | 28.9 ± 0.6 | 0.590 |
| Neutrophils |  | | | | |
| Relative (%) | 56.1 ± 2.1 | 59.2 ± 2.3 | 57.5 ± 2.0 | 57.9 ± 1.9 | 0.302 |
| Absolute (10^3^ uL^-1^) | 3.5 ± 0.3 | 3.6 ± 0.2 | 3.6 ± 0.2 | 3.6 ± 0.3 | 0.920 |
| Lymphocytes |  |  |  |  |  |
| Relative (%) | 30.2 ± 1.9 | 28.8 ± 2.1 | 30.6 ± 1.7 | 30.0 ± 1.8 | 0.727 |
| Absolute (10^3^ uL^-1^) | 1.8 ± 0.2 | 1.7 ± 0.1 | 1.8 ± 0.1 | 1.8 ± 0.1 | 0.470 |
| Monocytes |  | | | | |
| Relative (%) | 9.2 ± 0.6 | 8.1 ± 0.4 | 8.3 ± 0.4 | 8.4 ± 0.5 | 0.044 |
| Absolute (10^3^ uL^-1^) | 0.5 ± 0.03 | 0.5 ± 0.03 | 0.5 ± 0.03 | 0.5 ± 0.04 | 0.088 |
| Eosinophils |  | | | | |
| Relative (%) | 3.5 ± 0.7 | 3.0 ± 0.4 | 2.5 ± 0.3 | 2.7 ± 0.2 | 0.171 |
| Absolute (10^3^ uL^-1^) | 0.2 ± 0.03 | 0.2 ± 0.02 | 0.2 ± 0.02 | 0.2 ± 0.02 | 0.186 |
| Basophils |  | | | | |
| Relative (%) | 0.9 ± 0.1 | 0.8 ± 0.1 | 0.9 ± 0.1 | 0.9 ± 0.1 | 0.499 |
| Absolute (10^3^ uL^-1^) | 0.1 ± 0.05 | 0.1 ± 0.05 | 0.1 ± 0.03 | 0.1 ± 0.02 | 0.560 |
| Data are estimated means ± SE. Units in µM. Abbreviations: *Tx*, treatment, *HGB*, hemoglobin, *HCT*, hematocrit, *WBC*, white blood, *RBC*, red blood cell, *MCV*, mean corpuscular volume, *MCH*, mean corpuscular hemoglobin, *MCHC*, mean corpuscular hemoglobin concentration, *RDW*, red cell distribution width. | | | | | |

| **Table S4.** Comprehensive metabolic panel | | | | | |
| --- | --- | --- | --- | --- | --- |
|  | **Placebo** | | **NR** | | **Tx*Time** |
|  | **Baseline** | **Week 12** | **Baseline** | **Week 12** | **p-value** |
| Glucose (mg dL^-1^) | 91 ± 2 | 96 ± 2 | 103 ± 5 | 106 ± 6 | 0.507 |
| BUN (mg dL^-1^) | 18 ± 1 | 18 ± 1 | 16 ± 1 | 15 ± 1 | 0.503 |
| Creatinine (mg dL^-1^) | 1.0 ± 0.05 | 1.0 ± 0.05 | 0.9 ± 0.04 | 0.9 ± 0.04 | 0.487 |
| eGFR (mL min^-1^ 1.73m^-2^) | 70 ± 3 | 72 ± 4 | 78 ± 3 | 80 ± 3 | 0.612 |
| BUN:Creatinine | 18 ± 1 | 19 ± 1 | 18 ± 1 | 18 ± 1 | 0.322 |
| Sodium (mmol L^-1^) | 140 ± 0.3 | 141 ± 0.3 | 140 ± 0.5 | 141 ± 0.6 | 0.224 |
| Potassium (mmol L^-1^) | 4.3 ± 0.1 | 4.3 ± 0.1 | 4.3 ± 0.1 | 4.3 ± 0.1 | 0.222 |
| Chloride (mmol L^-1^) | 102 ± 0.5 | 103 ± 0.7 | 102 ± 0.4 | 103 ± 0.5 | 0.871 |
| CO_2_ (mmol L^-1^) | 25 ± 0.6 | 25 ± 0.6 | 24 ± 0.4 | 24 ± 0.5 | 0.333 |
| Calcium (mg dL^-1^) | 9.5 ± 0.1 | 9.5 ± 0.1 | 9.5 ± 0.1 | 9.5 ± 0.1 | 0.845 |
| Total Protein (g dL^-1^) | 7.1 ± 0.1 | 7.1 ± 0.1 | 6.9 ± 0.1 | 6.9 ± 0.1 | 0.504 |
| Albumin (g dL^-1^) | 4.4 ± 0.1 | 4.5 ± 0.1 | 4.5 ± 0.1 | 4.5 ± 0.1 | 0.560 |
| Globulin (g dL^-1^) | 2.6 ± 0.1 | 2.6 ± 0.1 | 2.4 ± 0.1 | 2.4 ± 0.1 | 0.761 |
| Albumin:Globulin | 1.7 ± 0.1 | 1.8 ± 0.1 | 1.9 ± 0.1 | 2.0 ± 0.1 | 0.948 |
| Bilirubin (mg dL^-1^) | 0.5 ± 0.1 | 0.5 ± 0.05 | 0.5 ± 0.04 | 0.5 ± 0.1 | 0.532 |
| Alk Phosphatase (IU L^-1^) | 74 ± 4 | 71 ± 4 | 73 ± 4 | 72 ± 3 | 0.691 |
| AST (SGOT) (IU L^-1^) | 21 ± 1 | 23 ± 1 | 21 ± 1 | 21 ± 1 | 0.138 |
| ALT (SGPT) (IU L^-1^) | 17 ± 1 | 19 ± 1 | 19 ± 2 | 18 ± 1 | 0.039 |
| Data are estimated means ± SE. Units in µM. Abbreviations: *Tx*, treatment, *BUN*, blood urea nitrogen, *eGFR*, estimated glomerular filtration rate, *CO_2_*, carbon dioxide, *alk*, alkaline, *AST*, aspartate aminotransferase, *ALT*, alanine phosphotransferase. | | | | | |

| **Table S5.** Total lipid panel | | | | | |
| --- | --- | --- | --- | --- | --- |
|  | **Placebo** | | **NR** | | **Tx*Time** |
|  | **Baseline** | **Week 12** | **Baseline** | **Week 12** | **p-value** |
| Total Cholesterol (mg dL^-1^) | 188 ± 7 | 192 ± 7 | 195 ± 6 | 196 ± 8 | 0.690 |
| Triglycerides (mg dL^-1^) | 111 ± 8 | 128 ± 20 | 119 ± 13 | 167 ± 63 | 0.589 |
| HDL (mg dL^-1^) | 61 ± 5 | 62 ± 6 | 65 ± 4 | 64 ± 4 | 0.513 |
| VLDL (mg dL^-1^) | 20 ± 1 | 23 ± 3 | 22 ± 2 | 20 ± 2 | 0.167 |
| LDL (mg dL^-1^) | 107 ± 6 | 108 ± 6 | 108 ± 5 | 109 ± 6 | 0.935 |
| Non-HDL (mg dL^-1^) | 127 ± 6 | 130 ± 5 | 129 ± 6 | 132 ± 8 | 0.910 |
| Data are estimated means ± SE. Units in µM. Abbreviations: *Tx*, treatment, *HDL*, high-density lipoprotein, *VLDL*, very low-density lipoprotein, *LDL*, low-density lipoprotein. | | | | | |

| **Table S6.** Treatment-emergent adverse events | | | | |
| --- | --- | --- | --- | --- |
|  | **Placebo** | | **NR** | |
| Term | Affected/ at Risk | # Events | Affected/ at Risk | # Events |
| Total | 12/25 (48%) | 13 | 1/24 (4.17%) |  |
| Cardiac |  |  |  |  |
| Heart palpitations | 1/25 (4%) | 1 | 0/24 (0%) | 0 |
| Gastrointestinal |  |  |  |  |
| Abdominal Pain | 0/25 (0%) | 0 | 1/24 (4.17%) | 2 |
| Vomiting | 1/25 (4%) | 1 | 0/24 (0%) | 0 |
| Diarrhea | 1/25 (4%) | 1 | 0/24 (0%) | 0 |
| Flatulence | 1/25 (4%) | 1 | 0/24 (0%) | 0 |
| Infections |  |  |  |  |
| COVID-19 | 0/25 (0%) | 0 | 1/24 (4.17%) | 1 |
| Urinary tract infection | 1/25 (4%) | 2 | 0/24 (0%) | 0 |
| Abnormal Labs |  |  |  |  |
| Creatinine increased | 0/25 (0%) | 0 | 1/24 (4.17%) | 1 |
| Triglycerides increased | 1/25 (4%) | 1 | 1/24 (4.17%) | 1 |
| Musculoskeletal |  |  |  |  |
| Chest wall pain | 2/25 (8%) | 2 | 0/24 (0%) | 0 |
| Neoplasms |  |  |  |  |
| Breast Cancer | 0/25 (0%) | 0 | 1/24 (4.17%) | 1 |
| Nervous system |  |  |  |  |
| Headache | 2/25 (8%) | 2 | 2/24 (8.33%) | 0 |
| Insomnia | 1/25 (4%) | 1 | 0/24 (0%) | 0 |
| Paresthesia | 1/25 (4%) | 1 | 0/24 (0%) | 0 |
| Psychiatric Disorders |  |  |  |  |
| Anxiety | 0/25 (0%) | 0 | 1/24 (4.17%) | 1 |
| Skin Disorders |  |  |  |  |
| Pruritus (from poison ivy) | 0/25 (0%) | 0 | 0/24 (0%) | 1 |
| Terminology from CTCAE Version 5.0 | | | | |
